# Association between inflammation-based prognostic markers and mortality after hip replacement

**DOI:** 10.1038/s41598-024-58646-y

**Published:** 2024-04-23

**Authors:** Ah Ran Oh, Ji-Hye Kwon, Gayoung Jin, So Myung Kong, Dong Jae Lee, Jungchan Park

**Affiliations:** Department of Anesthesiology and Pain Medicine, Samsung Medical Center, Sungkyunkwan University School of Medicine, 81 Irwon-Ro, Gangnam-Gu, Seoul, 06351 Korea

**Keywords:** Inflammation markers, Mortality, Hip replacement, Surgical outcomes, Prognostic markers, Biomarkers, Diagnostic markers, Predictive markers

## Abstract

We aimed to evaluate the association between inflammation-based prognostic markers and mortality after hip replacement. From March 2010 to June 2020, we identified 5,369 consecutive adult patients undergoing hip replacement with C-reactive protein (CRP), albumin, and complete blood count measured within six months before surgery. Receiver operating characteristic (ROC) curves were generated to evaluate predictabilities and estimate thresholds of CRP-to-albumin ratio (CAR), neutrophil-to-lymphocyte ratio (NLR), and platelet-to-lymphocyte ratio (PLR). Patients were divided according to threshold, and mortality risk was compared. The primary outcome was one-year mortality, and overall mortality was also analyzed. One-year mortality was 2.9%. Receiver operating characteristics analysis revealed areas under the curve of 0.838, 0.832, 0.701, and 0.732 for CAR, NLR, PLR, and modified Glasgow Prognostic Score, respectively. The estimated thresholds were 2.10, 3.16, and 11.77 for CAR, NLR, and PLR, respectively. According to the estimated threshold, high CAR and NLR were associated with higher one-year mortality after adjustment (1.0% vs. 11.7%; HR = 2.16; 95% CI 1.32–3.52; *p* = 0.002 for CAR and 0.8% vs. 9.6%; HR = 2.05; 95% CI 1.24–3.39; *p* = 0.01 for NLR), but PLR did not show a significant mortality increase (1.4% vs. 7.4%; HR = 1.12; 95% CI 0.77–1.63; *p* = 0.57). Our study demonstrated associations of preoperative levels of CAR and NLR with postoperative mortality in patients undergoing hip replacement. Our findings may be helpful in predicting mortality in patients undergoing hip replacement.

## Introduction

Hip replacement is a common procedure for treating hip fractures or disease such as osteoarthritis. Despite recent advances in surgical techniques and outcomes, mortality after hip replacement remains relatively high^1^. As the population of older adults continues to grow, so does the need for hip replacement surgeries^2^. The procedure is associated with a significant risk of mortality and various factors have been identified as contributing to this risk, such as age, sex, functional ability prior to the fracture, fracture type, type of surgery, pre-existing health conditions, length of hospital stay, low preoperative hemoglobin levels, and physical status score^3–5^. Given the role that systemic inflammatory response syndrome and patient frailty play in affecting outcomes after hip replacement surgery, inflammation-based markers and indicators of nutritional status in the preoperative period have the potential to provide useful insights into the preoperative management of the risk for postoperative mortality. These markers have the potential to provide a simple and objective way to assess the risk of mortality in the preoperative period and can help guide clinical decision-making.

These markers, which reflect systemic inflammatory response or nutritional condition, have been shown to be more effective in predicting outcomes when used in combination, such as the C-reactive protein (CRP)-to-albumin ratio (CAR), neutrophil-to-lymphocyte ratio (NLR), platelet-to-lymphocyte ratio (PLR), and modified Glasgow prognostic score (mGPS)^6–13^. In recent years, there has been an increasing interest in using inflammation-based markers as prognostic indicators for various diseases, including orthopedic diseases. These markers reflect the systemic response to inflammation, and changes in the numbers of different types of white blood cells have been found to be associated with the presence of systemic inflammation. These markers have been previously demonstrated to have predictive value in hip surgery^14–17^. Despite their potential usefulness, previous studies have mostly evaluated these markers individually, and not in combination.

In light of this, our study aimed to compare the predictive values of these markers in a cohort of patients who underwent hip replacement. By estimating the cut-off point of each biomarker, we divided the patients into different groups and compared the risk of postoperative mortality. Our findings offer insights into the usefulness of different markers in predicting the postoperative outcome of hip replacement. Our results may help clinicians to make more informed decisions about patient management and prognosis, using a simple index that combines multiple biomarkers.

## Materials and methods

Our study was a retrospective observational cohort study. Approval for this study was waived by the institutional review board at Samsung Medical Center, Seoul, Korea, because the data were extracted in de-identified form and the risk to study patients was minimal (SMC 2023–01-060). The requirement for written informed consent from individual patients was also waived by the institutional review board at Samsung Medical Center, Seoul, Korea. We conducted this research following the Declaration of Helsinki and reported our results in accordance with the Strengthening the Reporting of Observational Studies in Epidemiology guidelines.

### Study population and data collection

We enrolled adult patients who underwent hip replacement surgery at Samsung Medical Center between March 2010 and June 2020. Patients without available CRP, albumin level, and complete blood cell count within six months before surgery were excluded. Data were extracted in a de-identified form using the electronic archive system, “Clinical Data Warehouse Darwin-C,” which allows retrieval of data from electronic hospital records containing over 2.2 million surgeries, one billion lab results, 100 million disease codes, and 200 million prescriptions. Blood test results were automatically analyzed. Mortality data were regularly checked and updated using the National Population Registry of the Korea National Statistical Office to ensure no missing information on deaths. Medical records were reviewed by investigators who were unaware of patient mortality to prevent bias.

### Study outcomes and definitions

The primary study endpoint was mortality during one-year follow-up after surgery, and mortality during three-year follow-up was also compared.

Three prognostic indicators were determined using the following formulas: NLR = absolute neutrophil count / absolute lymphocyte count, PLR = absolute platelet count / absolute lymphocyte count, CAR = CRP / albumin. Additionally, mGPS was estimated from baseline CRP and albumin levels using the following calculation: Score 0: CRP ≤ 10 mg/L, Score 1: CRP > 10 mg/L and albumin ≥ 3.5 g/dL, and Score 2: CRP > 10 mg/L and albumin < 3.5 g/dL^18^. The Charlson comorbidity index was calculated using the 10^th^ revision of the International Statistical Classification of Diseases and Related Health Problems-10^19^.

### Statistical analysis

In this study, we used a number of statistical methods to analyze the data collected from their cohort of patients who underwent hip replacement. The categorical variables, such as the patients' sex, were presented in terms of numbers and percentages, while continuous variables, such as age and preoperative blood laboratory tests, were expressed as either means and standard deviations or medians and interquartile ranges, depending on the most appropriate measure of central tendency.

To compare the categorical variables, the researchers used the Chi-square test, and for continuous variables, we used the t-test or the Mann–Whitney test. To determine the optimal cut-off values of the biomarkers (CAR, NLR, and PLR) associated with one-year mortality, we performed receiver operating characteristics (ROC) curve analysis and calculated Youden's index. Then we compared the ROC curves using DeLong’s test^20^. After dividing the patients into low and high groups based on the estimated cut-off values, the researchers used Cox regression analysis to compare their mortalities, reporting the results as hazard ratios with 95% confidence intervals (CI). To reduce bias and achieve balance between groups, we conducted an adjustment using inverse probability weighting (IPW) with propensity scores for all relevant variables^21^. All statistical analysis was performed using R version 4.2.0, and a p-value less than 0.05 was considered statistically significant.

### Ethics approval and consent to participate

This retrospective cohort study was approved by the Institutional Review Board at Samsung Medical Center (SMC 2023–01-060). The requirement for written informed consent was waived because the data were collected retrospectively in de-identified form.

## Results

**Figure 1:**
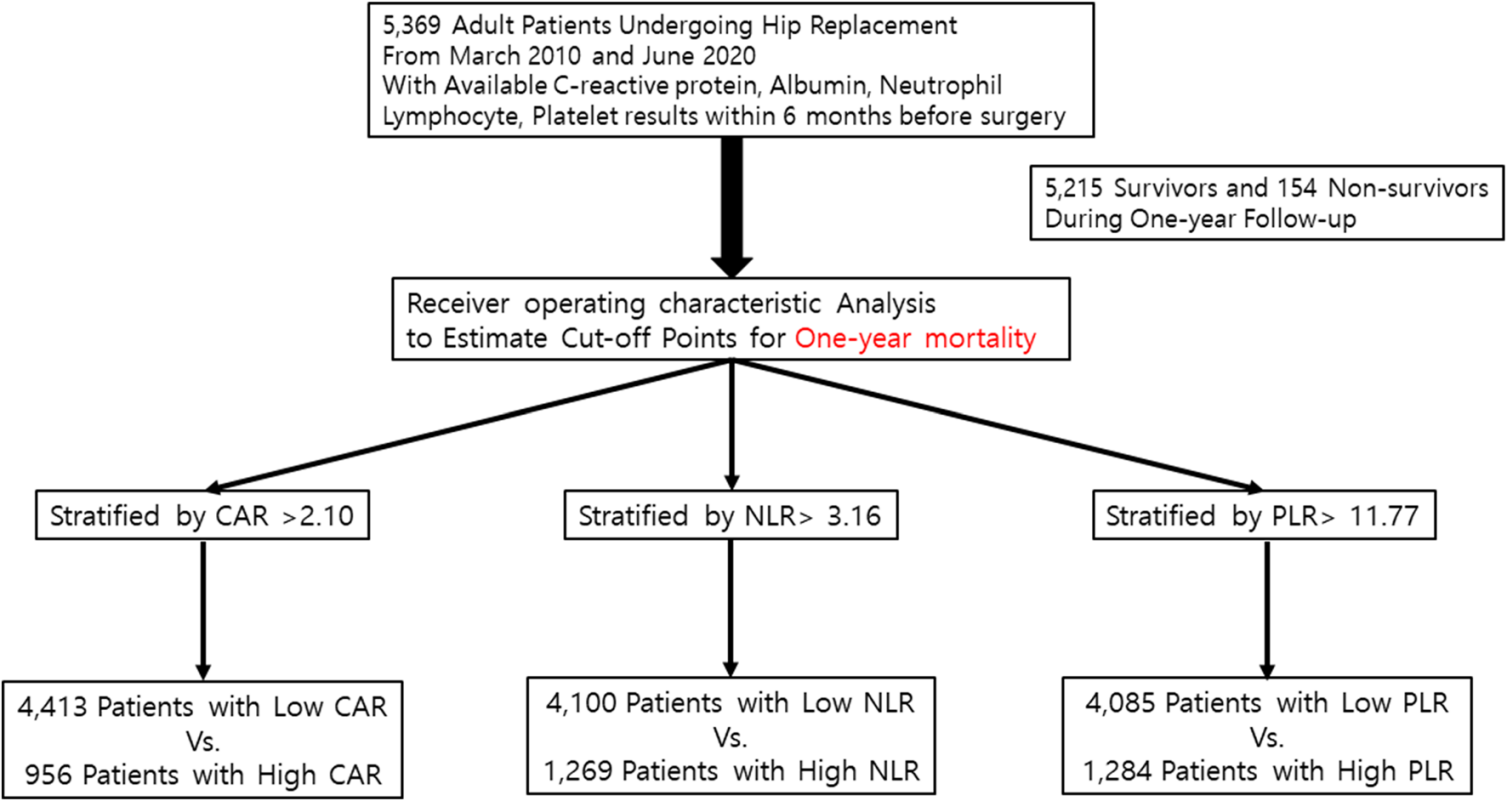
Study flowchart.

A total of 5,369 patients with available blood laboratory test results were included in the analysis. The overall study flowchart is shown in Fig. 1. Among them, 154 patients (2.9%) experienced one-year mortality. The Table 1 revealed notable differences between patients who experienced one-year mortality and those who did not. The former group tended to be older, predominantly male, and exhibited a higher prevalence of comorbidities. Additionally, emergency surgeries under general anesthesia were more frequent among patients with one-year mortality. Preoperative median values of CAR, NLR, PLR, and the proportion of patients with mGPS = 2 were significantly higher in the one-year mortality group.

**Table 1:** Baseline characteristics according to one-year mortality.

	Survivor (N = 5215)	Non-survivor (N = 154)	*p *value	*ASD*
C-reactive protein/albumin ratio	0.35 (0.13–1.07)	5.04 (1.59–14.76)	< 0.001	77.5
Neutrophil/lymphocyte ratio	1.91 (1.40–2.93)	5.32 (3.35–9.83)	< 0.001	99.4
Platelet/lymphocyte ratio	8.07 (6.17–11.33)	14.16 (8.09–19.81)	< 0.001	64.4
Modified Glasogow prognostic score			< 0.001	> 99
0	782 (15.0)	100 (64.9)		
1	4389 (84.2)	47 (30.5)		
2	44 (0.8)	7 (4.5)		
C-reactive protein, mg/l	1.50 (0.60–4.60)	15.90 (6.88–51.55)	< 0.001	78.7
Albumin, g/dl	4.40 (4.20–4.60)	3.60 (3.20–4.00)	< 0.001	> 99
Neutrophil	59.0 (52.0–67.3)	76.1 (68.0–83.7)	< 0.001	> 99
Lymphocyte	30.9 (23.0–37.5)	14.1 (8.1–20.2)	< 0.001	> 99
Platelet, K/mcL	242 (202–283)	191 (130–253)	< 0.001	51.5
*Age, years	59.2 (± 15.9)	69.7 (± 13.8)	< 0.001	70.8
*Body mass index	24.3 (± 3.7)	21.5 (± 3.5)	< 0.001	77.7
*Male	2217 (42.5)	76 (49.4)	0.11	13.8
Operative variables				
*Duration, minutes	74.7 (± 36.4)	92.9 (± 76.0)	< 0.001	30.5
*General anesthesia	713 (13.7)	90 (58.4)	< 0.001	> 99
*Total hip surgery	4609 (88.4)	22 (14.3)	< 0.001	> 99
*Emergency surgery	416 (8.0)	48 (31.2)	< 0.001	61.1
Habitual risk factor				
*Alcohol	1474 (28.3)	27 (17.5)	0.01	25.8
*Smoking	746 (14.3)	8 (5.2)	0.002	31.1
*Charlson comorbidity index	0.41 (± 1.10)	1.09 (± 1.93)	< 0.001	43.2
Myocardial infarction	18 (0.3)	2 (1.3)		
Heart failure	29 (0.6)	5 (3.2)		
Peripheral vascular disease	15 (0.3)	1 (0.6)		
Cerebrovascular disease	181 (3.5)	7 (4.5)		
Dementia	1 (0.0)	0		
Chronic pulmonary disease	2 (0.0)	1 (0.6)		
Rheumatic disease	160 (3.1)	4 (2.6)		
Peptic ulcer disease	3 (0.1)	0		
Mild liver disease	279 (5.3)	20 (13.0)		
Diabetes without complication	329 (6.3)	23 (14.9)		
Diabetes with complication	92 (1.8)	9 (5.8)		
Hemiplegia	23 (0.4)	2 (1.3)		
Renal disease	229 (4.4)	15 (9.7)		
Any malignancy	7 (0.1)	2 (1.3)		
Moderate to severe liver disease	12 (0.2)	4 (2.6)		
Metastatic solid tumor	0	0		
Human immunodeficiency virus	4 (0.1)	0		
Preoperative blood test				
*Hemoglobin, g/dL	13.2 (± 1.7)	11.2 (± 1.7)	< 0.001	> 99
*Creatinine, mg/dL	0.89 (± 0.71)	1.14 (± 1.30)	< 0.001	23.9

Values are *n* (%), mean (± standardized deviation), or median (interquartile range).

*ASD* absolute standardized mean difference, *IPW* inverse probability weighting.

*Following variables were retained for IPW adjustment.

**Figure 2:**
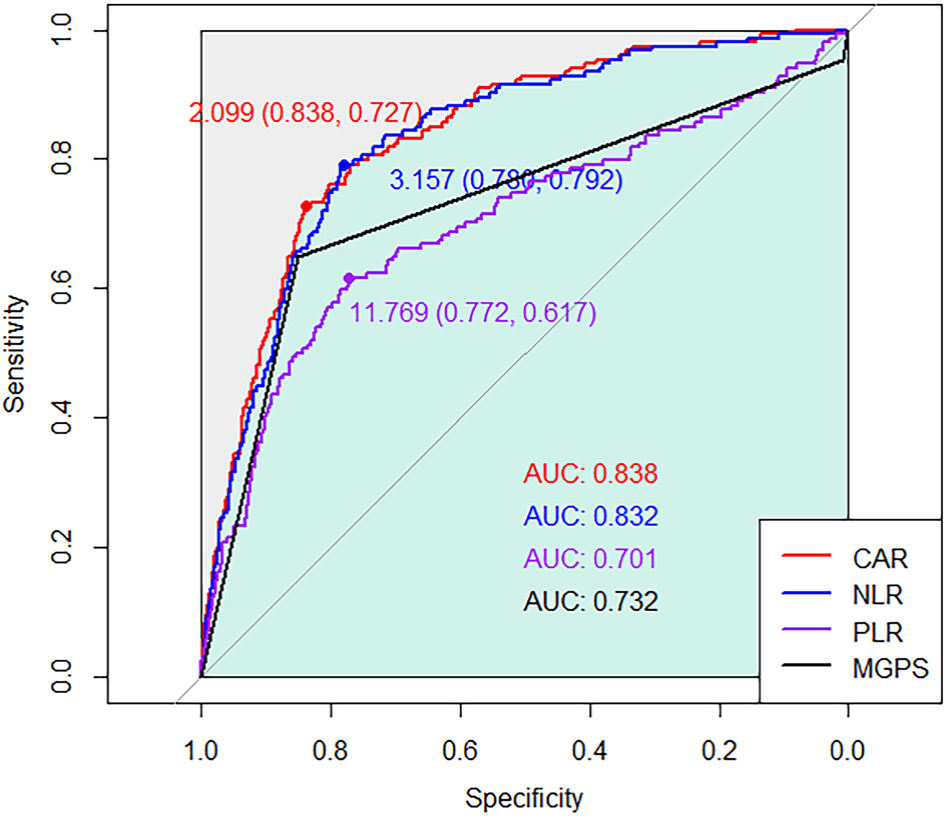
Receiver operating characteristics curves of one-year mortality for C-reactive protein-to-albumin ratio (CAR), neutrophil-to-lymphocyte ratio (NLR), platelet-to-lymphocyte ratio (PLR), and modified Glasgow prognostic score (mGPS).

ROC curves were constructed for each inflammatory marker, and the areas under the curve (AUCs) with 95% CIs for CAR, NLR, PLR, and mGPS were calculated as 0.838 (0.807–0.869), 0.832 (0.800–0.863), 0.701 (0.652–0.751), and 0.730 (0.688–0.776), respectively (Fig. 2). While the AUCs of CAR and NLR were comparable (z = 0.29; *p* = 0.77), the AUC of PLR was significantly lower than that of CAR (z = 4.96; *p* < 0.001) and NLR (z = 6.94; *p* < 0.001).

Optimal cut-off threshold values for one-year mortality were determined based on the maximum Youden's index: 2.10 for CAR, 3.16 for NLR, and 11.77 for PLR. Using these cut-off values, positive and negative predictive values were calculated for each marker. Subsequently, patients were classified into low and high groups for CAR (4,413 vs. 956), NLR (4,100 vs. 1,269), and PLR (4,085 vs. 1,284). Baseline characteristics were compared between these groups in Tables 2, 3, 4, revealing consistently higher values and greater incidence of relevant risk factors in the high groups.

**Table 2:** Baseline characteristics according to the estimated cut-off point of C-reactive protein/albumin ratio > 2.10.

	Low group (N = 4413)	High group (N = 956)	p-value	ASD before IPW	ASD After IPW
C-reactive protein/albumin ratio	0.25 (0.11–0.60)	6.51 (3.42–13.92)	< 0.001		
Neutrophil/lymphocyte ratio	1.77 (1.34–2.50)	4.06 (2.42–6.68)	< 0.001		
Platelet/lymphocyte ratio	7.63 (5.97–10.29)	12.55 (8.41–18.57)	< 0.001		
Modified Glasogow prognostic score			< 0.001		
0	1 (0.0)	881 (92.2)			
1	4372 (99.1)	64 (6.7)			
2	40 (0.9)	11 (1.2)			
C-reactive protein, mg/l	1.10 (0.50–2.60)	25.10 (13.60–49.73)	< 0.001		
Albumin, g/dl	4.50 (4.30–4.70)	3.80 (3.50–4.20)	< 0.001		
Neutrophil	57.6 (51.1–64.8)	71.9 (62.3–79.4)	< 0.001		
Lymphocyte	32.5 (25.7–38.5)	17.8 (11.7–25.7)	< 0.001		
Platelet, K/mcL	243 (205–282)	224 (169–286)	< 0.001		
*Age, years	57.4 (± 15.1)	69.2 (± 16.1)	< 0.001	75.8	0.3
*Body mass index	24.6 (± 3.6)	22.5 (± 3.8)	< 0.001	56.5	5.5
*Male	1895 (42.9)	398 (41.6)	0.48	2.7	1
Operative variables					
*Duration, minutes	75.3 (± 36.4)	75.0 (± 45.5)	0.85	0.6	4
*General anesthesia	476 (10.8)	327 (34.2)	< 0.001	58.4	2.5
*Total hip surgery	4123 (93.4)	508 (53.1)	< 0.001	> 99	< 0.1
*Emergency surgery	212 (4.8)	252 (26.4)	< 0.001	62.2	2.8
Habitual risk factor					
*Alcohol	1333 (30.2)	168 (17.6)	< 0.001	30	1.2
*Smoking	681 (15.4)	73 (7.6)	< 0.001	24.6	3.2
*Charlson comorbidity index	0.34 (± 0.97)	0.85 (± 1.65)	< 0.001	37.8	9.2
Myocardial infarction	12 (0.3)	8 (0.8)			
Heart failure	16 (0.4)	18 (1.9)			
Peripheral vascular disease	9 (0.2)	7 (0.7)			
Cerebrovascular disease	136 (3.1)	52 (5.4)			
Dementia	1 (0.0)	0			
Chronic pulmonary disease	2 (0.0)	1 (0.1)			
Rheumatic disease	116 (2.6)	48 (5.0)			
Peptic ulcer disease	2 (0.0)	1 (0.1)			
Mild liver disease	216 (4.9)	83 (8.7)			
Diabetes without complication	235 (5.3)	117 (12.2)			
Diabetes with complication	59 (1.3)	42 (4.4)			
Hemiplegia	17 (0.4)	8 (0.8)			
Renal disease	147 (3.3)	97 (10.1)			
Any malignancy	8 (0.2)	1 (0.1)			
Moderate to severe liver disease	6 (0.1)	10 (1.0)			
Metastatic solid tumor	0	0			
Human immunodeficiency virus	3 (0.1)	1 (0.1)			
Preoperative blood test					
*Hemoglobin, g/dL	13.4 (± 1.6)	11.9 (± 1.8)	< 0.001	93.1	0.2
*Creatinine, mg/dL	0.85 (± 0.57)	1.10 (± 1.22)	< 0.001	26	1.2

Values are *n* (%), mean (± standardized deviation), or median (interquartile range).

*ASD* absolute standardized mean difference, *IPW* inverse probability weighting.

*Following variables were retained for IPW adjustment.

**Table 3:** Baseline characteristics according to the estimated cut-off point of neutrophil/lymphocyte ratio > 3.16.

	Low group (N = 4100)	High group (N = 1269)	p-value	ASD before IPW	ASD after IPW
C-reactive protein/albumin ratio	0.27 (0.11–0.71)	1.93 (0.37–8.95)	< 0.001		
Neutrophil/lymphocyte ratio	1.67 (1.28–2.17)	5.45 (3.90–8.32)	< 0.001		
Platelet/lymphocyte ratio	7.20 (5.78–9.10)	15.85 (11.94–22.36)	< 0.001		
Modified Glasogow prognostic score			< 0.001		
0	306 (7.5)	576 (45.4)			
1	3776 (92.1)	660 (52.0)			
2	18 (0.4)	33 (2.6)			
C-reactive protein, mg/l	1.20 (0.50–3.10)	7.60 (1.50–33.00)	< 0.001		
Albumin, g/dl	4.50 (4.30–4.70)	4.00 (3.70–4.40)	< 0.001		
Neutrophil	56.1 (50.2–61.5)	77.1 (72.2–83.0)	< 0.001		
Lymphocyte	33.7 (28.3–39.2)	14.1 (10.0–18.3)	< 0.001		
Platelet, K/mcL	246 (208–285)	219 (173–272)	< 0.001		
*Age, years	56.2 (± 14.6)	70.0 (± 15.4)	< 0.001	91.7	9.8
*Body mass index	24.7 (± 3.6)	22.6 (± 3.6)	< 0.001	57.0	4.5
*Male	1838 (44.8)	455 (35.9)	< 0.001	18.4	7.8
Operative variables					
*Duration, minutes	76.3 (± 37.2)	71.9 (± 41.0)	< 0.001	11.1	7.8
*General anesthesia	416 (10.1)	387 (30.5)	< 0.001	52.3	6.3
*Total hip surgery	3922 (95.7)	709 (55.9)	< 0.001	> 99	6.7
*Emergency surgery	103 (2.5)	361 (28.4)	< 0.001	76.8	9.9
Habitual risk factor					
*Alcohol	1312 (32.0)	189 (14.9)	< 0.001	41.2	7.3
*Smoking	655 (16.0)	99 (7.8)	< 0.001	25.5	9.4
*Charlson comorbidity index	0.28 (± 0.86)	0.91 (± 1.68)	< 0.001	47	9.9
Myocardial infarction	12 (0.3)	8 (0.6)			
Heart failure	14 (0.3)	20 (1.6)			
Peripheral vascular disease	8 (0.2)	8 (0.6)			
Cerebrovascular disease	104 (2.5)	84 (6.6)			
Dementia	0	1 (0.1)			
Chronic pulmonary disease	0	3 (0.2)			
Rheumatic disease	99 (2.4)	65 (5.1)			
Peptic ulcer disease	1 (0.0)	2 (0.2)			
Mild liver disease	196 (4.8)	103 (8.1)			
Diabetes without complication	178 (4.3)	174 (13.7)			
Diabetes with complication	26 (0.6)	75 (5.9)			
Hemiplegia	15 (0.4)	10 (0.8)			
Renal disease	105 (2.6)	139 (11.0)			
Any malignancy	7 (0.2)	2 (0.2)			
Moderate to severe liver disease	6 (0.1)	10 (0.8)			
Metastatic solid tumor	0	0			
Human immunodeficiency virus	4 (0.1)	0			
Preoperative blood test					
*Hemoglobin, g/dL	13.5 (± 1.6)	12.2 (± 1.8)	< 0.001	74.5	13.3
*Creatinine, mg/dL	0.84 (± 0.52)	1.08 (± 1.17)	< 0.001	27	4.5

Values are n (%), mean (± standardized deviation), or median (interquartile range).

ASD, absolute standardized mean difference; IPW, inverse probability weighting.

*Following variables were retained for IPW adjustment.

**Table 4:** Baseline characteristics according to the estimated cut-off point of platelet/lymphocyte ratio > 11.77.

	Low group (*N* = 4085)	High group (*N* = 1284)	*p*-value	ASD before IPW	ASD after IPW
C-reactive protein/albumin ratio	0.27 (0.11–0.76)	1.16 (0.31–6.23)	< 0.001		
Neutrophil/lymphocyte ratio	1.67 (1.28–2.21)	5.00 (3.18–8.12)	< 0.001		
Platelet/lymphocyte ratio	7.13 (5.74–8.83)	16.12 (13.41–22.34)	< 0.001		
Modified glasogow prognostic score			< 0.001		
0	393 (9.6)	489 (38.1)			
1	3667 (89.8)	769 (59.9)			
2	25 (0.6)	26 (2.0)			
C-reactive protein, mg/l	1.20 (0.50–3.30)	4.80 (1.30–23.33)	< 0.001		
Albumin, g/dl	4.50 (4.20–4.60)	4.20 (3.80–4.50)	< 0.001		
Neutrophil	56.1 (50.2–61.9)	75.7 (68.3–82.7)	< 0.001		
Lymphocyte	33.7 (28.0–39.2)	15.1 (10.1–21.6)	< 0.001		
Platelet, K/mcL	235 (199–272)	269 (208–332)	< 0.001		
*Age, years	57.0 (± 14.8)	67.2 (± 16.8)	< 0.001	64.4	9.3
*Body mass index	24.6 (± 3.6)	22.9 (± 3.8)	< 0.001	45.4	3.4
*Male	1822 (44.6)	471 (36.7)	< 0.001	16.2	3
Operative variables					
*Duration, minutes	75.8 (± 37.3)	73.5 (± 41.0)	0.07	5.7	4.4
*General anesthesia	475 (11.6)	328 (25.5)	< 0.001	36.4	0.3
*Total hip surgery	3811 (93.3)	820 (63.9)	< 0.001	76.8	3.1
*Emergency surgery	175 (4.3)	289 (22.5)	< 0.001	55.5	3.9
Habitual risk factor					
*Alcohol	1259 (308)	242 (18.8)	< 0.001	28	2.7
*Smoking	613 (15.0)	141 (11.0)	< 0.001	12	4.4
*Charlson comorbidity index	0.34 (± 1.00)	0.72 (± 1.46)	< 0.001	29.8	0.1
Myocardial infarction	15 (0.4)	5 (0.4)			
Heart failure	20 (0.5)	14 (1.1)			
Peripheral vascular disease	9 (0.2)	7 (0.5)			
Cerebrovascular disease	109 (2.7)	79 (6.2)			
Dementia	0	1 (0.1)			
Chronic pulmonary disease	2 (0.0)	1 (0.1)			
Rheumatic disease	95 (2.3)	69 (5.4)			
Peptic ulcer disease	1 (0.0)	2 (0.2)			
Mild liver disease	223 (5.5)	76 (5.9)			
Diabetes without complication	200 (4.9)	152 (11.8)			
Diabetes with complication	45 (1.1)	56 (4.4)			
Hemiplegia	15 (0.4)	10 (0.8)			
Renal disease	140 (3.4)	104 (8.1)			
Any malignancy	8 (0.2)	1 (0.1)			
Moderate to severe liver disease	13 (0.3)	3 (0.2)			
Metastatic solid tumor	0	0			
Human immunodeficiency virus	4 (0.1)	0			
Preoperative blood test					
*Hemoglobin, g/dL	13.4 (± 1.7)	12.4 (± 1.7)	< 0.001	58.7	1.8
*Creatinine, mg/dL	0.87 (± 0.67)	0.98 (± 0.92)	< 0.001	13.5	0.3

Values are n (%), mean (± standardized deviation), or median (interquartile range).

*ASD* absolute standardized mean difference, *IPW* inverse probability weighting.

*Following variables were retained for IPW adjustment.

In terms of one-year mortality, the high CAR, NLR, and PLR groups exhibited substantially increased risks compared to their low counterparts (1.0% vs. 11.7%, HR = 13.50, 95% CI 9.47–19.35, *p* < 0.001 for CAR; 0.8% vs. 9.6%, HR = 13.31, 95% CI 9.02–19.64, *p* < 0.001 for NLR; 1.4% vs. 7.4%, HR = 5.44, 95% CI 3.93–7.53, *p* < 0.001 for PLR; Table 5). This relationship persisted for three-year mortality as well (2.0% vs. 16.8%, HR = 9.63, 95% CI 7.42–12.50, *p* < 0.001 for CAR; 1.8% vs. 13.9%, HR = 8.78, 95% CI 6.67–11.55, *p* < 0.001 for NLR; 2.6% vs. 11.0%, HR = 4.60, 95% CI 3.58–5.91, *p* < 0.001 for PLR).

**Table 5:** Mortalities according to the estimated thresholds C-reactive protein/albumin ratio > 2.10, neutrophil/lymphocyte ratio > 3.16, and platelet/lymphocyte ratio > 11.77.

	Low group	High group	Unadjusted HR (95% CI)	*p-*value	IPW adjusted HR	*p*-value
C-reactive protein/albumin ratio > 2.10	*N* = 4413	*N* = 956				
One-year mortality	42 (1.0)	112 (11.7)	13.50 (9.47–19.24)	< 0.001	4.64 (2.99–7.21)	< 0.001
Three-year mortality	87 (2.0)	161 (16.8)	9.63 (7.42–12.50)	< 0.001	2.95 (2.06–4.21)	< 0.001
30-day mortality	5 (0.1)	23 (2.4)	21.79 (8.28–57.32)	< 0.001	10.69 (3.47–32.92)	< 0.001
90-day mortality	10 (0.2)	49 (5.1)	23.84 (12.08–47.07)	< 0.001	9.37 (4.26–20.58)	< 0.001
Neutrophil/lymphocyte ratio > 3.16	*N* = 4100	*N* = 1269				
One-year mortality	32 (0.8)	122 (9.6)	13.31 (9.02–19.64)	< 0.001	5.31 (3.43–8.22)	< 0.001
Three-year mortality	72 (1.8)	176 (13.9)	8.78 (6.67–11.55)	< 0.001	2.97 (2.11–4.19)	< 0.001
30-day mortality	4 (0.1)	24 (1.9)	19.81 (6.87–57.10)	< 0.001	7.88 (2.53–24.56)	< 0.001
90-day mortality	6 (0.1)	53 (4.2)	29.75 (12.79–69.21)	< 0.001	11.36 (4.66–27.70)	< 0.001
Platelet/lymphocyte ratio > 11.77	*N* = 4085	*N* = 1284				
One-year mortality	59 (1.4)	95 (7.4)	5.44 (3.93–7.53)	< 0.001	2.05 (1.43–2.95)	< 0.001
Three-year mortality	107 (2.6)	141 (11.0)	4.60 (3.58–5.91)	< 0.001	1.58 (1.17–2.12)	0.002
30-day mortality	9 (0.2)	19 (1.5)	6.81 (3.08–15.05)	< 0.001	2.78 (1.18–6.54)	0.02
90-day mortality	19 (0.5)	40 (3.1)	6.90 (3.99–11.91)	< 0.001	2.68 (1.47–4.89)	0.001

*IPW* inverse probability weighting, *HR* hazard ratio, *CI* confidence interval.

IPW adjustment analysis retained age, male, hypertension, smoking, alcohol, Charlson comorbidity index, preoperative creatinine and hemoglobin levels, operative duration, general anesthesia, and type of surgery.

To ensure the robustness of our findings, we employed the IPW technique for adjustment, resulting in well-balanced variables across the groups (Tables 2, 3, 4). After adjustment, both high CAR and NLR remained significantly associated with an increased risk of one-year mortality (HR = 2.16, 95% CI 1.32–3.52, *p* = 0.002 for CAR; HR = 2.05, 95% CI 1.24–3.39, *p* = 0.01 for NLR), while PLR did not show a significant association (HR = 1.12, 95% CI 0.77–1.63, *p* = 0.57) (Table 5).

## Discussion

In our study, we investigated the relationship between various preoperative biomarkers and the risk of mortality after hip replacement surgery. The biomarkers that were evaluated were the CAR, NLR, and PLR. Our results showed that preoperative CAR and NLR were both strongly associated with higher mortality after hip replacement surgery. The predictive power of these two markers was found to be comparable, meaning that either one could effectively predict the risk of patient mortality. However, when patients were divided into groups based on their preoperative PLR values, no significant association was found between PLR and patient mortality. Overall, our study suggests that preoperative CAR and NLR may be valuable tools for predicting patient mortality after hip replacement surgery. The findings of this study may be useful for clinicians in making informed decisions about patient care and prognosis.

Inflammation is a crucial aspect of various diseases and has been shown to be a powerful predictor of patient outcomes in a wide range of medical conditions^6–13^. In the field of orthopedics, systemic inflammation has been linked to poor outcomes such as periprosthetic joint infections and surgical site infections^22,23^. To better understand the state of the systemic inflammatory response, various markers have been proposed and studied. White blood cells play a critical role in this response and previous studies have shown that the number of neutrophils in circulation increases while the number of monocytes and lymphocytes decreases during inflammation^24^. Additionally, low levels of lymphocytes are thought to indicate physiological stress and poor outcomes^25^. CRP is also a marker of acute-phase inflammatory response, and low level of albumin indicates malnutrition as well as inflammation severity and disease progression^26^. Infection, comorbidities, and malnutrition have been found to cause inflammation and decline in nutritional status, which is likely to be found in frail patients^26–28^. Together, a combination of these values can accurately reflect the mortality rate after hip replacement surgery, which is primarily performed on frail elderly patients. Our results showed that the estimated cut-off points had high negative predictive value, suggesting that these markers could be helpful in identifying those at relatively low risk and preserving the limited resources for other patients.

A key strength of this study lies in its accessibility and clinical relevance. By leveraging readily available blood markers from routine laboratory tests, we offer a practical approach for risk prediction without requiring additional specialized assays. Moreover, constructing composite markers from existing tests offers advantages in capturing the complex interplay between inflammation and mortality. Comparing the predictive performance of individual and combined markers, as assessed by AUC, highlights the potential of combining readily available data for improved risk stratification.

To further explore more intricate relationships between genetic and inflammatory markers, interaction prediction research in various fields of computational biology has provided promising avenues for future investigation. Techniques like deep learning-based models capable of identifying complex epistatic and non-additive interactions between genes and environment could offer valuable insights into personalized risk assessment for conditions with complex inflammatory involvement, including post-surgical outcomes. Notably, recent studies employing such approaches demonstrate the potential of these methods for unraveling intricate genetic interactions^29–35^. In addition, integrating our findings with future ordinary differential equation (ODE)-based models that incorporate specific inflammatory markers identified in this study could offer a deeper understanding of the dynamic interplay between inflammation and post-surgical outcomes^36,37^. By combining the strengths of readily available clinical data with the mechanistic insights provided by theoretical modeling, we can pave the way for more comprehensive risk prediction models and ultimately, the development of personalized preventative and therapeutic strategies.

According to PLR showed a lower predictive power compared with CAR and NLR, and patients with high PLR did not show increased risk of mortality after statistical adjustment. In contrast with our results, previous studies of patients undergoing hip surgery found a correlation between preoperative PLR and postoperative mortality^17,38^. However, despite these similarities, the study patients had some important differences from our patient population. Most previous studies evaluating the correlation between preoperative PLR and hip surgery outcomes have been conducted in elderly populations. However, our study included patients of all ages, with a relatively younger patient population compared to these previous studies. This difference in age range is significant as the influence of age on the outcome of hip replacement surgery is well documented. Additionally, the previous studies that evaluated the relationship between preoperative PLR and hip surgery outcomes also enrolled patients of all ages, but their patient populations were older compared to our study population. These differences in patient characteristics suggest that the results of our study may have a different implication for clinical practice compared to the results of these previous studies^17,38^.

Another factor to consider is that platelets may not be as sensitive a marker as the other biomarkers studied, and therefore may not be able to accurately reflect the health status of patients who are relatively healthier. This is supported by the results of a subgroup analysis from a previous study, which found that there was no significant correlation between preoperative PLR and hip surgery outcome in patients without anemia. This suggests that platelets may not be an ideal marker for these types of patients, and other markers may be more effective in predicting outcomes in this population^17^. It is also possible that there exists a publication bias, where only studies with positive results are published and reported. This phenomenon, known as the "file drawer problem," could potentially skew the interpretation of the results regarding the predictive value of PLR. Our study results imply that PLR may not be a universally effective predictor of patient outcomes after hip replacement surgery and that it should be used with caution. Further research is necessary to determine the specific patient populations for which PLR may have the greatest utility as a prognostic marker.

There are several limitations to consider when interpreting our study results. First, because this is a retrospective single-center study, our results may be influenced by unidentified factors, despite statistical adjustments. Second, the long study period may have introduced changes in surgical techniques and postoperative care that could have influenced results. Third, our study does not provide any treatment recommendations for patients with elevated biomarkers, and it is not possible to establish an optimal threshold from our analysis. Additionally, the positive predictive value of the markers was relatively low, indicating that they may not be as effective at identifying patients who are at high risk and should be used in conjunction with other clinical information. A prospective randomized trial is necessary to further validate these findings. Despite these limitations, this is the first study that comprehensively examines biomarkers in relation to mortality outcomes after hip replacement surgery.

In conclusion, our study demonstrated associations of preoperative levels of CAR and NLR with postoperative mortality in patients undergoing hip replacement. Our findings suggest that further studies are needed for these markers to be useful in identifying patients with high mortality risk and for developing strategies to improve outcomes after hip replacement surgery.

## Data Availability

All relevant data are available at reasonable request to the corresponding author (Jungchan Park).

## Abbreviations

CRP: C-reactive protein
CAR: C-reactive protein-to-albumin ratio
NLR: Neutrophil-to-lymphocyte ratio
PLR: Platelet-to-lymphocyte ratio
mGPS: Modified Glasgow prognostic score
ROC: Receiver operating characteristics
CI: Confidence intervals
IPW: Inverse probability weighting
